# Immunological Alterations due to Hemodialysis Might Interfere with Early Complications in Renal Transplantation

**DOI:** 10.1155/2019/8389765

**Published:** 2019-03-25

**Authors:** Kristin Mai, Andreas Boldt, Hans-Michael Hau, Michael Kirschfink, Stephan Schiekofer, Frieder Keller, Joachim Beige, Athanassios Giannis, Ulrich Sack, Franz Maximilian Rasche

**Affiliations:** ^1^Institute of Clinical Immunology, Medical Faculty, University of Leipzig, Leipzig, Germany; ^2^Department of Internal Medicine, Neurology and Dermatology, Clinic for Endocrinology and Nephrology, Section of Nephrology, University Hospital Leipzig, Leipzig, Germany; ^3^Department of Visceral, Transplantation, Vascular and Thoracic Surgery, University Hospital Leipzig, Leipzig, Germany; ^4^Institute of Immunology, University of Heidelberg, Heidelberg, Germany; ^5^Center for Geriatric Medicine, Bezirksklinikum Regensburg, Regensburg, Germany; ^6^Sigmund Freud PrivatUniversität, Wien, Österreich, Austria; ^7^Medical Department I, Nephrology Division, University Hospital Ulm, Ulm, Germany; ^8^Department of Nephrology, KfH Renal Unit, Hospital St. Georg, Leipzig, Germany; ^9^Martin-Luther-University Halle/Wittenberg, Halle, Germany; ^10^Institute for Organic Chemistry, University of Leipzig, Leipzig, Germany

## Abstract

**Background:**

Chronic or intercurrent alterations of the immune system in patients with end-stage renal disease (CKD) and intermittent hemodialysis (CKD5D, HD) have been attributed to an acute rejection of renal allograft.

**Methods:**

Leukocyte subsets in flow cytometry, complement activation, and concentrations of TGF*β*, sCD30 (ELISA), and interleukins (CBA) of fifteen patients eligible for renal transplantation were analyzed before, during, and after a regular HD.

**Results:**

Before HD, the median proportion of CD8+ effector cells, CD8+ CCR5+ effector cells, and HLA-DR+ regulatory T cells as well as the median concentration of soluble CD30 increased and naive CD8+ T cells decreased. During HD, there was a significant decrease in CD4- CD8- T cells (*p* < 0.001) and an increase in CD25+ T cells (*p* = 0.026), sCD30 (*p* < 0.001), HLA-DR+ regulatory T cells (*p* = 0.005), and regulatory T cells (*p* = 0.003). TGF*β* and sCD30 increased significantly over time. The activity of the classical complement pathway started to slightly increase after the first hour of HD and lasted until fifteen minutes after finishing dialysis. The decrease in the functional activity of the alternative pathway was only transient and was followed by a significant increase within 15 minutes after finishing the treatment.

**Conclusion:**

HD might interact with the allograft outcome by influencing T cell subsets and activation of the complement system in a biphasic course.

## 1. Introduction

Today, the dramatic descent of the frequencies of deceased kidney transplantations is determined by a decrease in the willingness for donating a deceased kidney and supported by strict political and legal conditions [1–4]. Therefore, the elimination of possible interfering or harming events to prevent acute rejections and to achieve an optimal conditioned organ in the highly complicated process of kidney transplantation is the main issue nowadays.

Modifiable and unmodifiable risk factors might contribute to the outcome of kidney graft function in the perioperative period in patients with chronic kidney disease (CKD) [5]. Hemodialysis (HD, CKD5D) immediately in the pre- or perioperative phase is supposed to be associated with a poorer renal allograft outcome due to nonimmunologic and immunologic effects [6]: (i) hemodynamic effects due to hypovolemia, vasoconstriction, and reduced renal plasma flow by activation of the renin-angiotensin-aldosterone system [7], (ii) activation of the immune system due to the contact, shear stress, or hydraulic pressure with the artificial surfaces and membranes [8–12], or (iii) alterations of the immune system due to chronic uremia [13–16].

In this prospective study, we aimed to characterize transient alterations of the innate and the adaptive immune system in response to new biocompatible HD devices, i.e., the newer polynephron membrane, and the permanent impact of chronic uremia (CKD).

## 2. Material and Methods

### 2.1. Inclusion and Exclusion Criteria, Study Protocol, and Materials

Between February and June 2015, fifteen patients were prospectively included who were on regular chronic intermittent HD using a high-flux dialyzer polynephron membrane (Nipro Elisio© 15H, Osaka, Japan) in the outpatient dialysis center, KfH Liebigstraße, Leipzig, Germany.

All patients were eligible for renal transplantation and listed in our transplant program. All patients gave their written informed consent to participate. The study protocol was approved by the ethics committee of the University of Leipzig (no. 365-14-17112014). Patients under 18 years or unable to give consent, patients with immunodeficiency or acute infection, patients with hemoglobin level below 10 g/dl, and pregnant or breastfeeding women were excluded.

Before, after one hour of, at the end of, and 15 minutes after a regular HD session, a 2.7 ml EDTA tube and a 4 ml whole blood tube were retrieved.

### 2.2. Blood Cell Count and Flow Cytometry

Two smears of each sample were stained with the kit “Hemacolor Schnellfärbung von Blutausstrichen” by Merck KGaA (Darmstadt, Germany). Subsequently, 100 leukocytes per object plate were analyzed for neutrophils and eosinophils using the microscope with a 100x optical magnification.

To determine the individual amounts of the different leukocytes, whole blood was screened with the panels—(i) “general lymphocyte overview,” (iv) “CD8 cell subsets,” and (v) “regulatory T cells”—as described by Boldt et al. [17] (see Table 1 in [17]).

125 *μ*l whole blood for each panel was incubated with the appropriate mixture of antibodies—for panel (i) 30 *μ*l and for panels (iv) and (v) 25 *μ*l—for 15 minutes. Subsequently, erythrocytes were lysed for 10 minutes using BD FACS Lysing Solution (BD Biosciences, Heidelberg, Germany). The specimens were centrifuged for 5 minutes at 500 g and then washed twice with phosphate-buffered saline. Afterwards, cells were analyzed on the BD FACSCanto II Flow Cytometer using the BD FACSDiva Software (BD Biosciences, Heidelberg, Germany).

### 2.3. ELISA and Multiplex Cytometric Bead Array

We used the following enzyme-linked immunosorbent assay (ELISA) following the description in the manufacturer's instructions: Human TGF*β*1 Quantikine ELISA (R&D Systems Minneapolis, MN 55413, USA) for the concentration of transforming growth factor- (TGF-) *β* and Human sCD30 ELISA (BioVendor Inc., Kassel, Germany) for the concentration of soluble CD30 as well as the Wieslab COMPL AP 330 kit and the Wieslab COMPL CP310 kit (Euro-Diagnostica, Malmö, Sweden) for the qualitative determination of alternative as well as classical complement pathway function.

IL-4, IL-6, IL-10, IL-17A, IFN-*γ*, and TNF-*α* were analyzed by a multiplex cytometric bead array using the BD CBA Human Soluble Protein Flex Systems (BD Biosciences, Heidelberg, Germany).

### 2.4. Statistical Analysis

All data are presented as median with range unless indicated otherwise. Statistical analysis was performed by using the Friedman test and the post hoc Wilcoxon signed-rank test (Sigma Plot version 11.0, San Jose, California, USA, and IBM SPSS 24, IBM, Armonk, NY 10540 USA). All results with *p* ≤ 0.05 were more closely reviewed. After Bonferroni correction (*n* = 15), all results with *p* ≤ 0.003 were considered to be significant. For comparison, lab internal reference ranges and reference ranges given in the manufacturer's instructions were used.

## 3. Results

### 3.1. Study Population, Leukocyte Count, and Influence of Ultrafiltration Rate on Hemoconcentration

Eight female and seven male patients with a median age of 49 years (min–max: 22 to 66 years) were treated—except for one person with two sessions per week—three times per week with chronic intermittent hemodialysis (Table 1, baseline characteristics).

The median of the ultrafiltration rate (UF) was 2100 ml (mean: 1990 ml, min–max: 0 to 3500 ml). There was no significant increase in eosinophil and neutrophil leukocytes in blood cell count in the Friedman and post hoc Wilcoxon tests in all patients and in separate analysis in regard to the groups with an UF higher or lower than 2100 ml (Tables 2 and 3).

### 3.2. Panel Reactive Antibodies, Cytokines, and Interleukins

In the Kruskal-Wallis test, there was no significant difference of all mentioned parameters in regard to the panel reactive antibodies (0-5%, 6-85%, and >85%) detected by Luminex technology (Immucor, Dreieich, Germany) routinely before transplantation.

Concentrations of cytokines and interleukins (TNF-*α*, IL-4, IL-6, IL-10, and IL-17) were not significantly altered during the study (Tables 2–4).

### 3.3. Leukocyte Subsets in FACS Analysis

The frequency of CD16+ CD56+ natural killer cells decreased temporarily within the first hour of dialysis from 9.1 to 7.3% (ref. 3 to 22%, *p* = 0.018). Subsequently, the median increased up to 11.6% (*p* = 0.018) from the second to the fourth sample. The percentage of CD3+ CD16+ natural killer T cells declined significantly analogous (1.9 to 1.6%, ref. 2.1 to 13.7%, *p* = 0.003) with no further remarkable increase. Furthermore, a significant constant decline in the median of double-negative T cells was observed over the entire observation period (4.2 to 3.4%, ref. 3 to 10.2%, *p* < 0.001).

CD4+ T lymphocytes significantly increased (*p* < 0.001) from the first to the second sampling (50.9 to 53.8%, ref. 31 to 51%, *p* < 0.001). However, CD8+ T lymphocytes decreased from 24.1 via 22.5% after one hour (ref. 18 to 35%, *p* = 0.005) to 20.8% at the end of the dialysis session (*p* = 0.035). There were no further remarkable changes over time for both T cell subpopulations.

**Figure 1:**
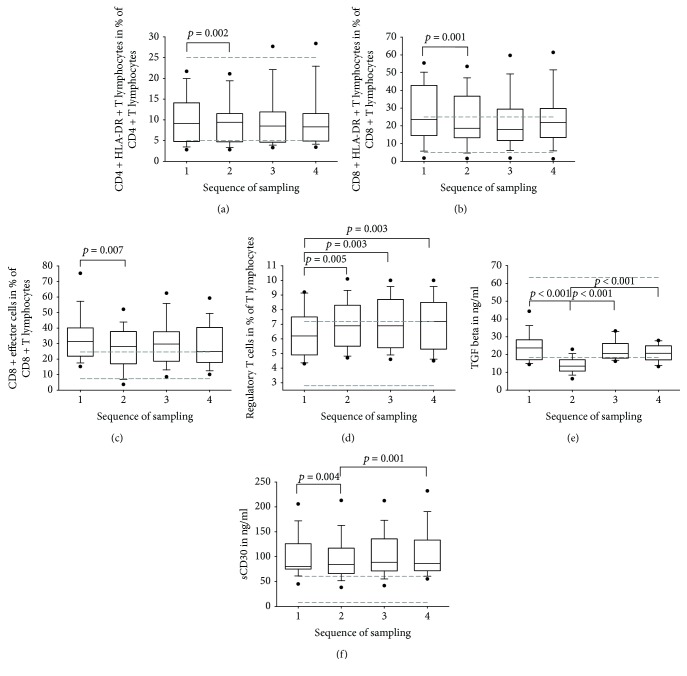
Box plots of flow cytometric analysis of the peripheral blood lymphocytes (a–d), TGF-beta (e), and sCD30 (f) before (1), after one hour of (2), at the end of (3), and 15 minutes after regular hemodialysis (4) in 15 patients eligible for renal transplantation. The dashed lines illustrate in each case the reference ranges. Wilcoxon signed-rank test: all results with *p* ≤ 0.05 are delineated; results with *p* ≤ 0.003 are considered to be significant (Bonferroni correction).

With respect to the early activation markers, the median of CD25+ T cells increased constantly during the HD (37.4 to 40.6%, ref. 22.9 to 44.9%, *p* = 0.018). Similar to CD4+ T cells, T helper cells expressing HLA-DR as a late activation marker increased significantly within the first hour of treatment (9.1 to 9.4%, ref. 5 to 25%, *p* = 0.002, Figure 1(a)). Interestingly, this increase was accompanied by a decrease in the percentage of CD8+ HLA-DR+ T cells (23.5 to 18.6%, ref. 5 to 25%, *p* = 0.001, Figure 1(b)). However, no further significant changes in the frequencies of both subsets could be seen.

The medians of the naive CD8+ T cells ranging from 16.5 to 22.1% were below the reference range 28.4-66.7%. No other relevant alterations for this subpopulation could be found over time. Irrespective of the decline from the first to the second sampling (32.1 to 28.1%, *p* = 0.007, Figure 1(c)), the percentages of CD8+ effector cells were above the appropriate reference values (7.4 to 24.6%) at any time. The medians of the frequencies of the effector cells expressing the C-C chemokine receptor type 5 (CCR5) were increased (ref. val.: <5.9%) and ranged from 6 to 10.5%. In the first hour, there was a decrease from 9.3 to 6% (*p* = 0.041) followed by an increase from the second to the fourth sample up to 10.5% (*p* = 0.007).

Furthermore, the percentage of regulatory T cells (Tregs) detected by the expression of surface antigens increased constantly and significantly during the observation time (6.2 to 7.2%, ref. 2.8 to 7.2%, *p* = 0.003, Figure 1(d)). Besides, the percentages of activated regulatory T cells (HLA-DR+) ranging from 25.1 to 28.2% were above the expected values (ref. 5.9 to 18.8%). Comparing the first with the fourth sample, the median increased characteristically (25.1 to 26.6%, *p* = 0.005).

However, in this study there was no significant change of CD19+ B cells, CD4+ CD8+ T cells, CD3+ T cells, CD4+ CD38+ T cells, CD8+ CD38+ T cells, CD8+ central memory T cells, CD8+ effector memory T cells, CD8+ CCR5+ effector memory T cells, TH17 cells, naive Tregs, and regulatory memory T cells.

### 3.4. TGF*β* and sCD30 Levels

TGF*β* was in the lower reference range (18.3-63.7 ng/ml) except for the second sample taken after one hour of HD wherein the concentration was significantly decreased from 23.7 to 13.5 ng/ml (*p* < 0.001). Subsequently, the concentration increased significantly from 13.5 to 20.7 ng/ml over time (*p* < 0.001, Tables 3 and 4 and Figure 1(e)).

The sCD30 level was always above the reference range (7.7-60.5 ng/ml) revealing a significant increase at each period from the first to the second sample (80.1 to 84.5 ng/ml, *p* = 0.004) and from the second to the fourth sample (84.5 to 86 ng/ml, *p* < 0.001, Tables 3 and 4 and Figure 1(f)).

### 3.5. Complement System

The levels of complement activity were within the reference ranges for the classical (median min–max: 101.7 to 106.4%, ref. 69 to 129%) as well as the alternative pathway (median min–max: 78.2 to 87.9%, ref. 30 to 113%).

The activity of the classical pathway started to slightly increase after one hour of HD and lasted until fifteen minutes after finishing dialysis (101.7 to 103.9%, *p* = 0.003). The decrease in the functional activity of the alternative pathway (indicating complement activation with consumption) during the dialysis session itself (86.7 to 78.2%, *p* = 0.03) was only transient and followed by a significant increase up to 87.9% within 15 minutes after finishing the treatment (*p* = 0.003).

There were no significant differences in statistics if data were calculated for the sample size (*n* = 10) excluding patients with former renal transplantation (four with lost graft function—three patients received mycophenolate mofetil 500 twice per day, one patient 5 mg prednisolone—and one patient with graft nephrectomy).

## 4. Discussion

**Figure 2:**
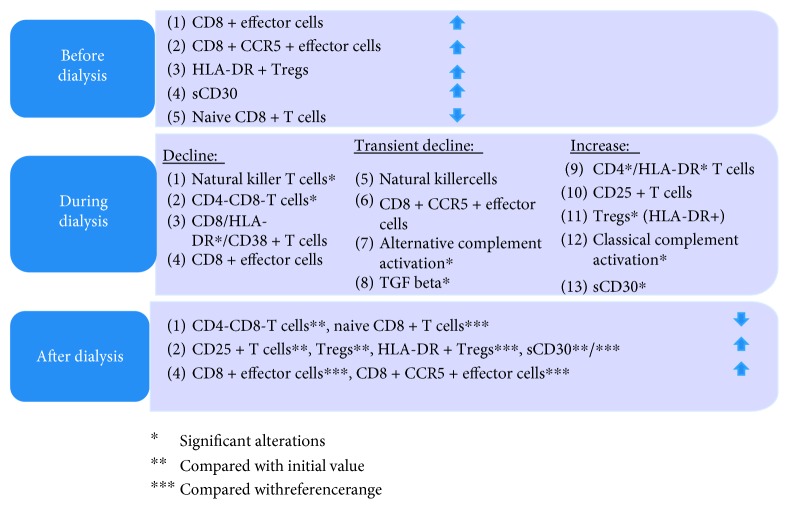
Synopsis of immunologic alterations in patients with hemodialysis. ^∗^Significant alterations; ^∗∗^compared with the initial value; ^∗∗∗^compared with the reference range.

In this prospective study, we examined for the first time the relevant immunologic effects induced by HD with newer polynephron membranes which may have an impact on the outcome of renal allografts (Figure 2 and Table 5 [10–14, 16, 18]).

In regard to lymphocytes, some of the previously described effects could not be found in our patients. Effects could be found in our samples neither for TH1 and TH2 cells nor for TH17 cells [9, 19]. This could be a consequence of our gating strategy, subdividing cells thoroughly and maybe missing some effects this way. Other findings were as expected. We demonstrated an increase in the frequencies of CD8+ effector cells, CD8+ CCR5+ effector cells, HLA-DR+ regulatory T cells, and sCD30 serum concentration as well as a decrease in the frequency of naïve CD8+ T cells in peripheral blood of CKD5D patients eligible for transplantation compared to the reference ranges (Figure 2 and Table 5).

For the first time, we found a biphasic course with either a temporary decrease in natural killer cells, CD8+ HLA-DR+ T cells, complement alternative pathway function, and concentration of TGF*β* or going along with a temporary increase in CD4+ T cells and CD4+ HLA-DR+ T cells during one single dialysis session. The biphasic course possibly related to biocoating of the membrane regarding the amphiphilic nature of the polyether sulfone membrane material [20] during the first one to two hours might be important for the posttransplantation process. Obviously, aromatic groups are able to interact with several biomolecules containing thiol, hydroxyl, carboxyl, and amino groups to name only a few [21]. The shift of the leukocyte subsets might be related to the cytokine release initiated by the uncoated membrane and could be explained by a release phenomenon from homing compartments of the lymphoid tissue [22].

As previously described [23–25], we also observed a significant increase in sCD30 serum concentration and an increase in the frequency of regulatory T cells as well as a decrease in double-negative T cells.

In general, the results presented in the literature concerning the composition of peripheral blood leukocytes in CKD5D patients are contradictory—Daichou et al. reported a higher percentages of CD8+ T cells compared with healthy controls [19], Weimer et al. described a reduced CD8+ T cell counts [26], and Bergström et al. could not find differences in the frequency of CD8+ T cells [27].

At first, we demonstrate a decreased relative proportion of naïve CD8+ T cells and increased frequencies of CD8+ effector cells and HLA-DR+ Tregs in CKD5D patients before HD. Consistent with Litjens et al. [28], we also found increased frequencies of CD8+ CCR5+ T lymphocytes.

We found for the first time a decline of natural killer T cells and of natural killer cells within the first hour of treatment. A reduction of the natural killer cells was only described in cellulose-triacetate membrane [14].

Double-negative T cells (CD4- CD8-) are proinflammatory cells in autoimmune diseases and are associated with the tolerance induction [29]. Lower frequencies are described in uremic patients [30]. In our study, the percentages of CD4- CD8- T cells were already close to the lower reference range before HD and further decreased during HD.

Graft rejection is considered to be the consequence of an adaptive immune response mainly based on T lymphocytes, i.e., CD4+ T cells, against foreign major histocompatibility complexes [31]. However, the role of CD8+ T cells has not been finally clarified [32]. In addition, an increase in activation markers on T cells, CD25 and HLA-DR+, is associated with transplant rejection [33].

In the first hour, we detected an increase in CD4+ T cells and activated CD4+ HLA-DR+ T cells. This increase was accompanied by a decrease in frequencies of CD8+ T cells and CD8+ HLA-DR+ T cells. Also, the frequencies of CD3+ T cells expressing the early activation marker CD25 were increased over the whole observation period.

In regard to the CD8+ T cell subsets, there was a decrease in naive T cells during HD. This might be based on activation and subsequent differentiation to effector or memory cells after the contact with the filter. Furthermore, we observed an increase in the frequencies of the CD8+ effector cells and CD8+ effector cells which expresses CCR5 on the cell surface that is crucial for the migration of lymphocytes into the foci of inflammation [34] and is associated with loss of graft function [28]. However, Szczepańska et al. could not show any differences in the frequency of CD8+ CCR5+ T lymphocytes neither in comparison with healthy controls nor in the course of one single dialysis session in children with HD [15].

The percentages of Tregs increased significantly during HD but remained within the normal range. Furthermore, there was a trend to increase in the frequency of the activated Tregs during HD. In summary, there was an increase in cells that in general are considered responsible for graft rejection (CD4+ T cells, CD4+ HLA-DR+ T cells, CD3+ CD25+ T cells, CD8+ effector cells, and CD8+ CCR5+ T cells) but also a slight increase in cells that might induce tolerance (Tregs, HLA-DR+ Tregs).

We also observed a biphasic course with a decrease in the functional activity of the classical complement activation within the first hour of treatment as well as of the alternative pathway during dialysis, indicating a transient but only moderate complement activation. This minor effect results from better biocompatibility of nowadays applied membranes [10, 18]. Therefore, it is not surprising that our data ranged within the reference ranges.

Transforming growth factor beta is associated with the differentiation of T cells to Tregs but also with chronic allograft rejection [16, 35]. As previously described by Stefoni et al. [16], we also found lower TGF*β* levels in dialyzed patients—with a marked reduction after 30 minutes of treatment with a return to basal values three hours after finishing dialysis.

Activated T cells cleave sCD30 from the cell membrane [23, 36], and it is supposed that high preoperative [23, 24, 36–38] and postoperative [25, 39, 40] concentrations are associated with allograft loss, i.e., in concentrations more than 100 U/ml [23–25]. In accordance with the literature [41–43], sCD30 concentrations were elevated and further increased during the dialysis session.

It could be suspected that the three physical principles of hemodialysis filtration, convection, osmosis, and diffusion may contribute to the observed changes of immunological parameters in our study. However, the ultrafiltration rate in our patients was comparatively low with a median of two liters [44] and no significant changes in neutrophil or eosinophil cell count were described in our patients. Therefore, significant effects of hemoconcentration on cells and molecules, i.e., macromolecules (>60 kDa: CRP, sCD30, and complement factors) or convection of middle size molecules (500-60000 kDa: TGF*β*, TNF-*α*, IL-4, IL-6, IL-10, and IL-17), could be neglected in our study population and no correction in regard to the ultrafiltration rate was performed [44–46]. Elimination of the middle molecules is supplied by the kidneys in patients with renal excretion, i.e., steady state, or by convection in hemodialysis. However, 60 percent of the patients had a residual excretion of more than 500 ml and a significant decrease was not observed generally in all measured molecules with a comparable molecular weight. In contrast, the serum concentration of TGF*β* increased during the study.

## 5. Conclusions

In CKD5D patients of our study, frequencies of CD8+ effector cells, CD8+ CCR5+ effector cells, and HLA-DR+ regulatory T cells were increased and frequencies of naïve CD8+ T cells were decreased. The concentration of sCD30—a biomarker used to predict rejection—was elevated in our patients and further increased during HD. HD increased regulatory T cells, decreased CD4- CD8- T cells, and causes a biphasic course with either a transient decline (natural killer T cells, CD8+ HLA-DR+ T lymphocytes, activation of the complement system, and higher concentration of TGF*β*) or a transient increase (CD4+ T lymphocytes, CD4+ HLA-DR+ T lymphocytes). The results of our data suppose that in the first hours after the beginning of the HD intercurrent changes will be remarkable. This normalizes until the fourth hour. Therefore, we argue not to consider short-time dialysis before kidney transplantation. Further studies will be necessary with a detailed focus on the complement system, biocoating, and a follow-up of these patients after renal transplantation.

## Figures and Tables

**Table 1:** Baseline characteristics.

Patients	Sex category (m: male/f: female)	Age (years)	Blood group^1^	Kidney disease^2^	HbA1c (%)	C-reactive protein (g/l)	Time on dialysis (months)	Cumulative weekly dialysis dose (hours)	Anticoagulation^3^	Kt/*V*	Residual diuresis^4^	Serum creatinine (*μ*mol/l)	Blood urea nitrogen (BUN) (mmol/l)	Anti-Hbs antibodies (mIU/ml)^5^	Number of previous transplantations	Panel reactive antibodies (%)
1	m	32	A (+)	2	5.7	7.1	55	14.25	UFH	1.24	0	836	12.32	1	1	4
2	f	66	0 (+)	0	5.1	13.1	60	8	UFH	1.43	1	418	10.22	2	0	0
3	m	37	A (+)	0	5.3	19	47	14	UFH	1.22	1	1123	10.59	2	0	0
4	m	36	0 (-)	2	5.2	2	144	15	LMWH	1.11	0	1296	12.41	2	0	0
5	m	46	A (+)	1	5	8.5	142	13	LMWH	1.59	0	1275	14.98	0	1	0
6	m	61	A (+)	0	5.8	13.6	27	12	UFH	1.02	1	778	7.84	2	0	0
7	m	55	0 (-)	0	4.8	12.9	97	14	LMWH	1.54	1	1193	14.42	0	0	0
8	f	22	A (+)	0	5.2	1.2	45	12	UFH	1.86	0	1049	11.57	2	0	0
9	f	49	0 (-)	0	4.9	3	88	15	UFH	2.14	1	871	10.13	2	0	18
10	f	57	A (+)	0	4.8	2.7	48	13	Ar	1.49	0	1061	9.66	2	0	25
11	m	28	A (-)	0	4.6	8.9	44	15	UFH	1.12	0	1469	12.65	2	1	47
12	f	60	B (+)	3	4.5	4.3	45	15	UFH	1.84	1	223	2.29	0	0	70
13	f	53	B (+)	0	5.2	21.8	111	14	UFH	1.74	1	964	11.43	1	1	78
14	f	38	0 (+)	3	8.9	1	33	12	UFH	1.26	0	849	9.24	0	0	78
15	f	56	B (+)	2	5.6	0.9	68	13	UFH	1.61	0	1069	13.63	2	1	85
Median		49			5.2	7.1	55	14		1.49		1049	11.43			9

*n* = 15. 14 Caucasians except patient no. 15 (Vietnamese). Furthermore, all patients received treatment with iron and recombinant erythropoietin. ^1^Blood group: (-): Rhesus negative; (+): Rhesus positive. ^2^0: analgetics, cisplatine, ischaemia, orellanus, nephrosclerosis, pyelonephritis, CKD5D (unknown reasons), shrinkle kidneys, adult dominant polycystic kidney disease. 1: Alport syndrome; 2: focal segmental glomerulosclerosis; 3: diabetes mellitus. ^3^UFH: unfractionated heparin; LMWH: low molecular weight heparin; Ar: argatroban. ^4^0: ≤500 ml; 1: >500 ml. ^5^0: <10; 1: 10-100; 2: >100.

**Table 2:** Leucocyte subsets before, during, and after hemodialysis.

	Before dialysis	After one hour of dialysis	At the end of dialysis	15 minutes after end of dialysis	Reference range
Natural killer cells (% of lymphocytes)					
Median (min–max)	9.1 (3.7-22.1)	7.3 (3-19.8)	11.5 (3.8-19)	11.6 (3.4-21.6)	3-22
Natural killer T cells (% of lymphocytes)					
Median (min–max)	1.9 (0.4-8.6)	1.6 (0.1-5.1)	1.9 (0.4-5.9)	2.1 (0.3-7.1)	2.1-13.7
CD4+ T cells (% of lymphocytes)					
Median (min–max)	50.9 (36.8-66.5)	53.8 (39.5-68.8)	52.3 (43.2-64.3)	51.7 (42.6-64.5)	31-51
CD8+ T cells (% of lymphocytes)					
Median (min–max)	24.1 (13.6-41.4)	22.5 (13.5-27.2)	20.8 (11.6-33.8)	22.2 (11.8-31)	18-35
CD4- CD8- T cells (% of T cells)					
Median (min–max)	4.2 (1.9-7.7)	3.7 (1.6-6.3)	3.3 (1.5-5.4)	3.4 (1.7-6.5)	3-10.2
CD25+ T cells (% of T cells)					
Median (min–max)	37.4 (24.4-55)	38.6 (26.9-54.8)	40.6 (27.4-59.1)	39.5 (26.8-56.4)	22.9-44.9
CD4+ HLA-DR+ T cells (% of CD4+ T cells)					
Median (min–max)	9.1 (2.8-21.7)	9.4 (2.8-21.1)	8.5 (3.3-27.7)	8.3 (3.4-28.4)	5-25
CD8+ HLA-DR+ T cells (% of CD8+ T cells)					
Median (min–max)	23.5 (1.8-55.4)	18.6 (1.6-53.3)	18 (1.8-59.7)	21.9 (1.4-61.4)	5-25
Naive CD8+ T cells (% of CD8+ T cells)					
Median (min–max)	16.5 (8.5-43.7)	22.1 (11-53.2)	16.6 (6.8-46.4)	18.7 (6.8-56.5)	28.4-66.7
CD8+ effector cells (% of CD8+ T cells)					
Median (min–max)	31.2 (15.2-75.3)	28.1 (3.7-52.1)	29.7 (8.6-62.5)	30.9 (10.1-59.3)	7.4-24.6
CD8+ CCR5+ effector cells (% of CD8+ T cells)					
Median (min–max)	9.3 (0.2-29.9)	6 (0.7-26)	10.4 (1.2-25.6)	10.5 (1-31.5)	< 5.9
Regulatory T cells (% of T cells)					
Median (min–max)	6.2 (4.3-9.2)	6.9 (4.7-10.1)	6.9 (4.6-10)	7.2 (4.5-10)	2.8-7.2
HLA-DR+ Tregs (% of Tregs)					
Median (min–max)	25.1 (9.3-38.4)	28.2 (9.5-38.5)	25.7 (8.6-45.8)	26.6 (9.4-46)	5.9-18.8
Eosinophils (% of leukocytes)					
Median (min–max)	4 (1-10)	3 (0-7)	3 (0-6)	2 (0-6)	1-3
Neutrophils (% of leukocytes)					
Median (min–max)	63 (51-77)	68 (46-87)	68 (52-79)	67 (50-87)	55-75

Median (min (minimum)–max (maximum)) in percent in fluorescence-activated cell sorting analysis (FACS) except for neutrophil and eosinophil counts (differential blood count by microscopy). Statistical analysis with nonparametric tests are shown in Table 3.

**Table 3:** Synopsis of the significant alterations in the immune system before (1), during (2), at the end (3), and 15 minutes after hemodialysis (4) of Tables 2 and 4.

	Friedman	Wilcoxon signed-rank test					
Period sample	1 to 4	1 and 2	1 and 3	1 and 4	2 and 3	2 and 4	1 and 4
Natural killer cells	0.028	0.018	0.524	0.524	0.121	0.018	0.389
Natural killer T cells	0.003	0.003^∗^	0.08	0.194	0.268	0.058	0.233
Double-negative T cells	<0.001	<0.001^∗^	0.005	<0.001^∗^	0.077	0.524	0.244
CD4+ T cells	0.001	<0.001^∗^	0.121	0.107	0.151	0.095	0.599
CD8+ T cells	0.01	0.005	0.035	0.055	0.208	0.978	0.208
CD25+ T cells	0.006	0.042	0.018	0.026	0.229	0.229	0.978
CD4+ HLA-DR+ T cells	0.039	0.002^∗^	0.454	0.561	0.169	0.173	0.303
CD8+ HLA-DR+ T cells	0.016	0.001^∗^	0.432	0.169	0.89	0.151	0.389
CD8+ naive T cells	0.165^∗^	—	—	—	—	—	—
CD8+ effector cells	0.013	0.007	0.804	0.561	0.121	0.073	0.524
CD8+ CCR5+ T cells	0.001	0.041	0.454	0.169	0.083	0.007	0.208
Regulatory T cells	0.006	0.005	0.003^∗^	0.003^∗^	0.584	0.359	0.588
HLA-DR+ Tregs	0.05	0.847	0.151	0.005	0.169	0.055	0.083
Complement CP	0.046	0.073	0.847	0.359	0.121	0.003^∗^	0.296
Complement AP	0.017	0.153	0.03	0.679	0.454	0.073	0.003^∗^
TGF*β*	<0.001	<0.001^∗^	0.72	0.359	<0.001^∗^	<0.001^∗^	0.524
sCD30	0.004	0.004	0.454	0.561	0.107	<0.001^∗^	0.326
Eosinophils	0.062^∗^	—	—	—	—	—	—
Neutrophils	0.744^∗^	—	—	—	—	—	—

Blood samples: before (1), after one hour (2), at the end (3), and 15 minutes after the end of HD (4). Statistical analysis with the Friedman test and the post hoc Wilcoxon signed-rank test. ^∗^*p* values ≤ 0.003 were considered to be significant according to Bonferroni. CP: complement activation, classical pathway; AP: complement activation, alternative pathway. ^∗^Not tested due to a nonsignificant Friedman test.

**Table 4:** TGF*β* level, sCD30 level, and complement activation before, during, and after hemodialysis.

	Before dialysis	After one hour of dialysis	At the end of dialysis	15 minutes after end of dialysis	Reference range^∗^
TGF*β* (ng/ml)					
Median (min–max)	23.71 (14.4-44.34)	13.47 (6.38-22.92)	20.58 (16.19-23.25)	20.68 (13.24-27.89)	18.3-63.4
sCD30 (ng/ml)					
Median (min–max)	80.1 (45.05-205.88)	84.45 (38.18-212.96)	88.67 (41.75-212.47)	85.96 (55.2-232.18)	7.7-60.5
Classical pathway (%)					
Median (min–max)	106.4 (86.7-124.8)	101.7 (80.6-115)	104.4 (80.8-114.6)	103.9 (85.5-118.5)	69-129
Alternative pathway (%)					
Median (min–max)	86.7 (54.4-115)	84.5 (49.8-98.4)	78.2 (41.8-94.8)	87.9 (63.9-104.8)	30-113

^∗^Mentioned in the manufacturer's instructions (Human TGF-*β*1 Quantikine ELISA, R&D Systems; Human sCD30 ELISA, BioVendor; Wieslab COMPL CP310, Euro-Diagnostica; Wieslab COMPL AP 330, Euro-Diagnostica). min: minimum; max: maximum. Statistical analysis with nonparametric tests are shown in Table 3.

**Table 5:** Intradialytic alterations of the immune system.

	Alterations during HD	Literature (comments)	*n*	Membrane
Natural killer cells	Frequencies ↓	Grooteman et al. [14]	8	CM, CA, PS
		*Present publication* (transient)	15	PN
Natural killer T cells	Frequencies ↓	*Present publication* (transient)	15	PN
CD4+ T lymphocytes	Frequencies ↑	Grooteman et al. [14]	8	CM, CA, PS
		*Present publication*	15	PN
		Szczepańska et al. [15]	12	
CD8+ T lymphocytes	Frequencies =	Grooteman et al. [14]	8	PS
	Frequencies ↓	Grooteman et al. [14]	8	CM, CA
		*Present publication*	15	PN
	Number ↓	Yoon et al. [13]	21	
Activation marker (CD25, HLA-DR)	Frequencies =	Grooteman et al. [14]	8	CM, CA, PS
	Frequencies ↑	*Present publication* (CD25, CD4+ HLA-DR+ T cells)	15	PN
	Frequencies ↓	*Present publication* (CD8+ HLA-DR+ T cells)	15	PN
Regulatory T cells	Frequencies ↑	*Present publication*	15	PN
Complement activation	Transient increase within 15 minutes	Girndt et al. [18] (MAC)	14	PA, H
	Transient increase in C3a within 30 minutes	Rousseau et al. [11]	6	PS, CA, CU
		Varela et al. [12]	12	CM, H, MC
		Hörl et al. [10] (all: CU > S)		
	Transient increase in C5a within 60 minutes	Varela et al. [12]	12	CM, H, MC
	Transient decrease within 60 minutes	*Present publication* (alternative & classical pathway)	15	PN
TGF*β*	Transient decrease in concentration	*Present publication*	15	PN
		Stefoni et al. [16]	155	H, PAN, PS
sCD30	Concentration ↑	*Present publication*	15	PN

Abbreviations: *n*: number of dialyzed patients. Membrane: CA: cellulose acetate; CM: cuprammonium; CU: cuprophan; H: Hemophan; MC: modified cellulose; PA: polyamide; PAN: polyacrylnitrile; PN: polynephron; PS: polysulphone. References: Grooteman et al. [14], Szczepańska et al. [15], Yoon et al. [13], Girndt et al. [18], Rousseau et al. [11], Varela et al. [12], Hörl [10], and Stefoni et al. [16].

## Data Availability

The datasets used and/or analyzed during the current study are available from the corresponding author on reasonable request at any time.

## Abbreviations

CCR5: C-C chemokine receptor type 5
CKD5D: Chronic kidney disease stage 5 dialysis
EDTA: Ethylenediamine tetraacetic acid
ELISA: Enzyme-linked immunosorbent assay
HD: Hemodialysis
IL: Interleukin
max: Maximum
min: Minimum
PRA: Panel reactive antibodies
ref. range: Reference value
sCD30: Soluble CD30
TGF: Transforming growth factor
Tregs: Regulatory T cells
UF: Ultrafiltration.
